# An early warning system to forecast the close of the spring burning window from satellite-observed greenness

**DOI:** 10.1038/s41598-017-14730-0

**Published:** 2017-10-27

**Authors:** Paul D. Pickell, Nicholas C. Coops, Colin J. Ferster, Christopher W. Bater, Karen D. Blouin, Mike D. Flannigan, Jinkai Zhang

**Affiliations:** 1 Department of Forest Resources Management, University of British Columbia, Vancouver, Canada; 2 Alberta Agriculture and Forestry, Edmonton, Canada; 3 Department of Renewable Resources, University of Alberta, Edmonton, Canada

## Abstract

Spring represents the peak of human-caused wildfire events in populated boreal forests, resulting in catastrophic loss of property and human life. Human-caused wildfire risk is anticipated to increase in northern forests as fuels become drier, on average, under warming climate scenarios and as population density increases within formerly remote regions. We investigated springtime human-caused wildfire risk derived from satellite-observed vegetation greenness in the early part of the growing season, a period of increased ignition and wildfire spread potential from snow melt to vegetation green-up with the aim of developing an early warning wildfire risk system. The initial system was developed for 392,856 km^2^ of forested lands with satellite observations available prior to the start of the official wildfire season and predicted peak human-caused wildfire activity with 10-day accuracy for 76% of wildfire-protected lands by March 22. The early warning system could have significant utility as a cost-effective solution for wildfire managers to prioritize the deployment of wildfire protection resources in wildfire-prone landscapes across boreal-dominated ecosystems of North America, Europe, and Russia using open access Earth observations.

## Introduction

The importance of changing wildfire regimes under a changing climate has become a major focus of research and management efforts globally^1^. In the boreal forests of North America it is anticipated that climate warming will result in increases in fuel availability and wildfire severity^2^ with concomitant increases in wildfire danger and wildfire season length^3^, particularly for the circumboreal forest^4^. The effects of increasing human activity such as urbanization and resource extraction within boreal forests remain poorly understood with respect to potential human-caused wildfire ignitions and wildfire risk^5,6^. Humans ignited approximately 57% of all wildfires in Alberta, accounting for 63% of annual area burned (~200,000 ha) between 1996 and 2014^7^. More than two-thirds of human-caused wildfires in Alberta occur during what is recognized across Canada as the spring burning window^8^, a period of increased ignition and wildfire spread potential spanning the time between the disappearance of snow and vegetation green-up. The spring burning window is most pronounced in Alberta, which ends when understory and overstory vegetation produce fresh leaves that are high in moisture content, reducing the potential of wildfire ignition and spread^8^. Alberta is a model jurisdiction for investigating human-caused wildfire risk during the springtime due to the confluence of human ignition sources and a seasonal change in fuel moisture modulated by climate and leaf phenology. For these reasons, May is the most active wildfire month resulting in the highest monthly area burned in Alberta^7^, and springtime burning is common to circumboreal ecosystems across North America, Europe, and Russia^9–11^.

Previous research has shown that the moisture content in fine fuels is a critical risk factor of human-caused wildfire activity^12,13^. Fine fuels with diameters smaller than 2.5 cm tend to respond rapidly to abrupt changes in weather conditions (i.e., temperature, wind speed, relative humidity, and precipitation) due to a high surface area to volume ratio that increases rates of evapotranspiration compared with larger fuels^14^. Current models of fuel moisture, such as the Canadian Forest Fire Danger Rating System, integrate wildfire weather data with empirical knowledge of evapotranspiration rates for different forest fuel types to estimate daily fuel availability and potential wildfire behavior^14^. These models are ledgers of fuel moisture that capture moisture content of fine fuels and large coarse fuels including deep soils and duff, accounting for daily wetting and drying as well as seasonal drying factors^15^. The Canadian system has been broadly adopted around the world, including across Europe, however similar systems of wildfire danger rating exist such as the National Fire Danger Rating System in the United States^16^ and the MacArthur Forest Fire Danger Index in Australia^17^. Although these systems are widely used around the world to estimate wildfire danger and wildfire behavior, the weather-based inputs are not direct measurements of fuel moisture, and the Canadian system makes broad assumptions about the homogeneity of fuel types. As a result, there remains unaccounted spatial variability in the estimate of wildfire behavior within and between wildfires from these indirect fuel moisture models^18^.

Satellite observations of vegetation greenness have been successfully used to estimate wildfire danger codes and fuel moisture codes for northern boreal forests^19,20^. Satellite observations from the Moderate Resolution Imaging Spectroradiometer (MODIS) provide a unique opportunity to observe and monitor fuel moisture from the flush of new vegetation, since healthy leaf area has long been shown to have a strong relationship with spectral greenness indices and leaf moisture^21^. Since leaf area is a proxy for foliar fine fuel moisture^8^ and is readily measured directly from satellite-derived greenness indices, fewer assumptions are required about site-specific fuel types when assessing wildfire spread potential. Moreover, using cloud-free composites of MODIS imagery, surface reflectance observations are available nearly every eight days at a spatial resolution of 250 m for the entire planet^22^. Thus, the spectral, spatial, and temporal resolutions of the satellite observations are ideal for monitoring the spring burning window as expressed by vegetation greenness for large areas.

In this paper we propose that satellite-observed greenness trends will be useful for estimating the peak of the spring burning window for any location, thus forming an early warning system for forecasting human-caused wildfire activity, which has wide application in forested ecosystems beyond the focus site. To do so, we investigated the relationship between fine temporal changes in satellite-derived greenness, and the commencement of the spring burning window as quantified by human wildfire ignitions in Alberta’s Forest Protection Area, the forested areas where wildfires are actively managed using provincial resources. To facilitate the development of the early warning system, we provide an assessment of the model error (in days) as a function of day-of-year of observation acquisition and show ground conditions of modelled predictions using a ground network of phenology cameras. The developed relationships of the spring burning window can augment existing decision support systems by providing near real-time proxy information of fuel moisture to wildfire managers for allocating suppression resources and planning prescribed burns.

## Methods

### Study area

**Figure 1:**
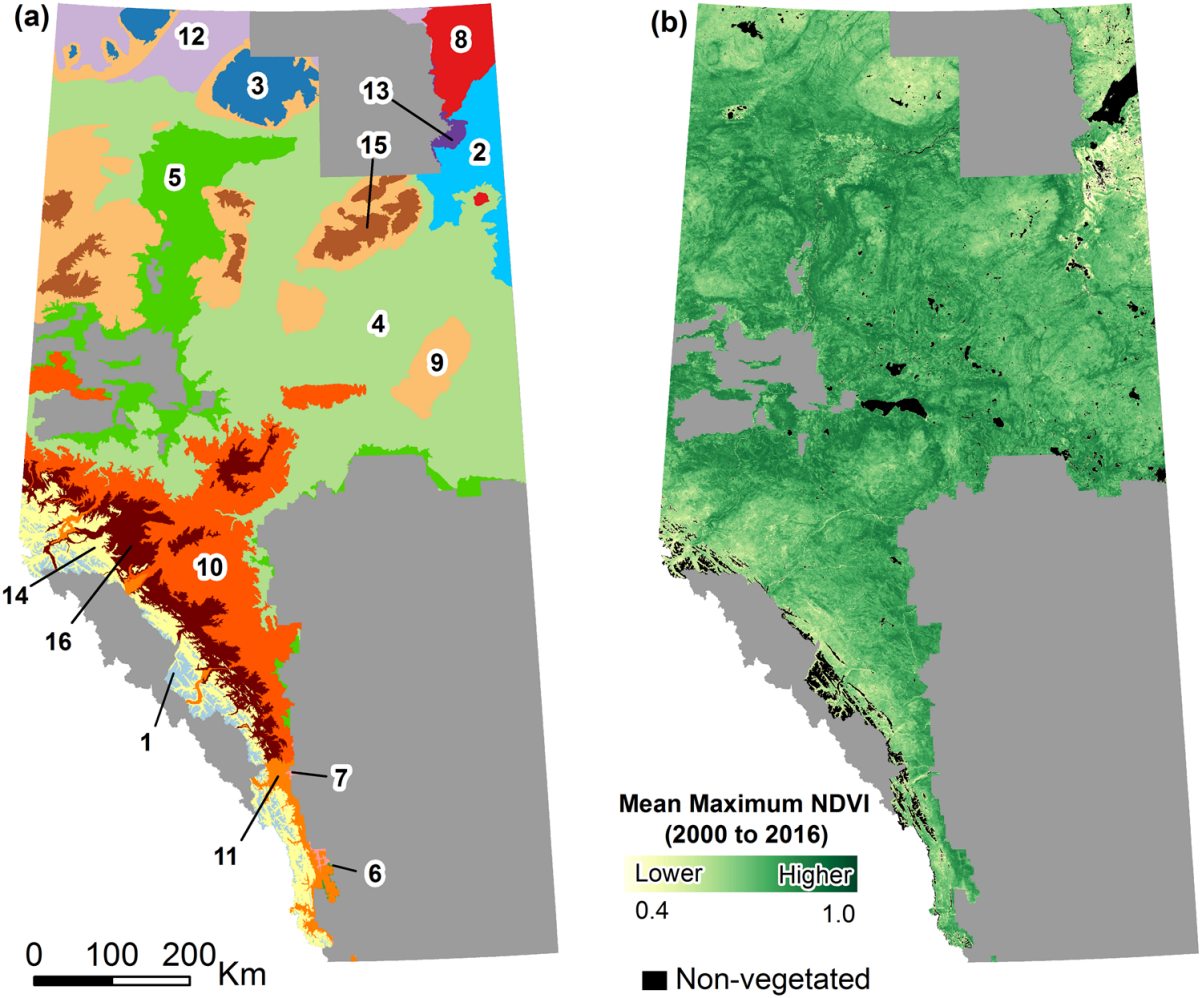
(**a**) Map of natural sub-regions within Alberta’s wildfire protection area: (1) Alpine; (2) Athabasca Plain; (3) Boreal Subarctic; (4) Central Mixedwood; (5) Dry Mixedwood; (6) Foothills Fescue; (7) Foothills Parkland; (8) Kazan Uplands; (9) Lower Boreal Highlands; (10) Lower Foothills; (11) Montane; (12) Northern Mixedwood; (13) Peace-Athabasca Delta; (14) Subalpine; (15) Upper Boreal Highlands; (16) Upper Foothills. (**b**) Mean maximum greenness as expressed by the Normalized Vegetation Differenced Index (NDVI) for years 2000 to 2016. Maps created using ArcMap 10.5.1. (http://desktop.arcgis.com/en/arcmap/).

The province of Alberta comprises 661,848 km^2^ of land and freshwater ecosystems^23^, of which approximately 42% is considered to be forested^24^. The provincial government actively manages a wildfire protection area totaling 392,856 km^2^ or approximately 59% of the province^25^. The wildfire protection area spans 16 natural sub-regions with varying continental and interior climates (Fig. 1a). Growing degree days >5 °C ranges from 317 in the Alpine to 1,388 in the Foothills Fescue and the mean date of last spring frost ranges from May 27 (day-of-year [DOY] 147) in the Athabasca Plain and Peace-Athabasca Delta to July 13 (DOY 194) in the Alpine^26^. Topography of the region is highly variable with elevations ranging from 150 m in the Northern Mixedwoods and Kazan Uplands to 3,650 m in the Alpine^26^. Mean annual precipitation ranges from 377 mm in the Peace-Athabasca Delta to 989 mm in the Alpine, of which, 68% and 48% falls during the growing season, respectively^26^. Approximately 74% of the wildfire protection area is considered boreal forest, dominated by pure and mixedwood stands of *Populus tremuloides*, *Populus balsamifera*, *Picea mariana*, *Picea glauca*, *Pinus contorta*, *Pinus banksiana*, and *Larix laricina*. Distribution of various vegetation types are determined climatically by precipitation and minimum annual temperature (*i.e*., cold hardiness), which vary along the large latitude and elevation gradients that dominate the province.

### Human-caused wildfire ignitions data

Wildfire ignitions for Alberta were retrieved from the historical wildfire database for the years 2000 to 2014^7^. The historical wildfire database contains wildfire ignitions observed from a variety of sources including lookout observers, forest officers, the public (310-FIRE), wildfire patrols, aircraft, wildfire crews, infrared scans, and satellite imagery. Attributes are recorded for each wildfire ignition including the estimated date of ignition, the cause of ignition, and wildfire size after being extinguished. Small wildfire ignitions (<0.1 ha) likely represented illegal campfires and were removed from the dataset. Ignitions caused by lightning or without sufficient information on the date of occurrence were also removed. The remaining human-caused wildfire ignitions between 2000 and 2014 totaled *N* = 5,325 and accounted for approximately 45% of the total area burned represented in the database and approximately 92% of human-caused area burned. The number of human-caused ignitions that occurred between February 26 (DOY 57) and July 15 (DOY 196) totaled *n* = 4,017 or approximately 75% of all human-caused wildfire ignitions across all calendar years.

### Modelling human-caused wildfire ignitions

Recent studies have used lightning ignition data to estimate wildfire risk during the wildfire season^27,28^. However, wildfire risk is known to vary according to ignition source and season^29^. For example, it has been postulated that deciduous leaf phenology in the western Canadian interior forest provides a major climate feedback on seasonal patterns of temperature and precipitation, with the possible conclusion that evapotranspiration from the leaf-on forest is at least partly responsible for the development of thunderstorms in the region^30^. Thus, lightning ignitions are not a suitable proxy for human-caused wildfire activity during leaf-off conditions, and local environmental conditions such as rapid springtime greening of vegetation, time since last snow cover (or some other suitable representation of the moisture content of deep, compact organic layers), and human-caused wildfire ignitions also need to be considered. The timing of vegetation greening shifts inter-annually, however vegetation greening has been shown to be relatively stable in terms of amplitude^31^. By contrast, it is challenging to know *a priori* when and where humans ignite wildfires or what weather conditions will be prevalent beyond several weeks. Therefore, our approach was first to relate the timing of human-caused ignitions to vegetation green-up and then use the satellite remote sensing data to predict green-up dates as an indicator of peak springtime human-caused wildfire risk.

Human-caused wildfire risk was modelled by converting wildfire ignition counts to wildfire days, defined as any day of the calendar year when one or more human-caused wildfire ignitions are reported. A wildfire day is a meaningful concept to wildfire managers who require a prediction of whether a given location will require wildfire protection resources on a given day and provides a well-established, robust statistical random variable for binomial modelling^32^. The probability of a human-caused wildfire day during a given year may be estimated as a function of a Poisson distribution, which approximates the binomial process, where the number of wildfire starts occurring on any given DOY for all available years 2000–2014 from the historical wildfire database is the rate parameter λ^12^.

### Satellite observations

The MODIS sensor onboard the Aqua and Terra satellites acquires imagery in the red and near infrared spectral wavelengths for most northern latitudes on a daily basis at 250 m spatial resolution. We retrieved eight-day surface reflectance image composites generated for the MOD09Q1 Version 6 product between February 26 (DOY 57) and July 12 (DOY 193) for years 2000 to 2016^22^. The eight-day MODIS composites represented a compromise between obtaining sufficient observations to track vegetation phenology over a given season while minimizing data gaps caused by clouds and atmospheric conditions^22^. For every year, a total of 18 eight-day composite MODIS images were available. The MODIS images were downloaded directly from the United States Geological Survey^22^, converted to a 10 degree North American Datum 1983 Transverse Mercator projection, and mosaicked to cover the province of Alberta. Wildfire managers in Alberta use broad ecological natural sub-regions to manage wildfire protection resources, so we adopted those regions for summarizing trends of satellite-derived greenness across the wildfire protection area of the province^26^.

### Modelling vegetation green-up

Greenness was estimated for each eight-day MODIS composite using the normalized difference vegetation index (NDVI)^21,33^. We first excluded low-quality observations using the quality control data distributed with the MOD09Q1 product. The annual NDVI curves were averaged to derive a mean greenness normal for each DOY and then each season between 2000 and 2016 was screened for noise by converting the annual NDVI observations to a z-score. Observations were flagged as noise if they exceeded the one-sided upper tail critical value at α = 0.10. Assessing the upper tail ensured that observations with significantly lower greenness than expected from the 17-year average were flagged as noise since these observations were likely related to error in atmospheric correction procedure. For each year from 2000 to 2016, a one-dimensional cubic spline was fit^34^ to the seasonal NDVI values for each MODIS pixel to temporally interpolate the 8-day MODIS composites to daily estimated values of NDVI. Green-up was defined as the date when the temporally interpolated NDVI curve reached the mean of the minimum and maximum NDVI values for the season, also known as the half-maximum^35^. This procedure resulted in a spatially-explicit estimation of vegetation green-up across the region. A mask was applied to extract pixels occurring within the wildfire protection area of Alberta and green-up statistics were further summarized by natural sub-regions (Fig. 1a). Vegetation productivity varies considerably across the wildfire protection area and the mean maximum vegetation greenness as expressed by NDVI is shown in Fig. 1b for the years 2000 to 2016. Green-up was not estimated for seasonal NDVI pixel series with more than five noisy observations since this degree of missing data can result in inaccurate curve fitting. Additionally, our initial analyses showed that the maximum NDVI value is most likely observed in the last two dates of our seasonal series, so if those dates were noisy then we did not estimate green-up.

### Forecasting vegetation green-up

We tested the ability of the half-maximum greenness system to accurately forecast the peak spring burning window by simulating real-time availability of satellite observations throughout each annual season. This was achieved by sequentially replacing the 2000–2016 mean NDVI values with actual satellite observations for any given day-of-year and then recalculating the green-up date using the new greenness curve. The greenness curves that emerged from concatenating actual greenness observations with the mean greenness normal were then used to estimate the green-up date for the cumulative set of satellite observations {NDVI_i=0_, … NDVI_n_} in a year, where *i* indexes the observation position in the year out of *n* = 18 total observations in any year. The mean absolute error (in days) between the observed and the forecasted green-up DOY from the cubic spline model was calculated for each pixel and for each satellite observation replaced sequentially in the season. This process was repeated for all years and days-of-year, up to and including July 4 (DOY 185).

### Validating vegetation green-up

A network of 16 phenological cameras were installed *in situ* in the Foothills, Subalpine, and Alpine natural sub-regions during the growing season between April and October in 2009^36–38^. The cameras acquired five red-green-blue photos daily over an hour at noon local standard time to compensate for varying shadow and illumination effects in the scene. From these sets of images, we show some examples of the forest condition on or around the dates that we predict high probability of a wildfire day based on the half-maximum green-up model.

### Data availability

Alberta wildfire ignitions are publicly available from http://wildwildfire.alberta.ca/resources/historical-data/spatial-wildwildfire-data.aspxwildfirewildfirewildfirewildfire. The 8-day surface reflectance MODIS products (MOD09Q1) are publicly available from http://e4ftl01.cr.usgs.gov/MOLT/MOD09Q1.006/. All processed data and results including estimations of vegetation green-up and forecast simulations are available from the corresponding author upon reasonable request.

## Results

**Figure 2:**
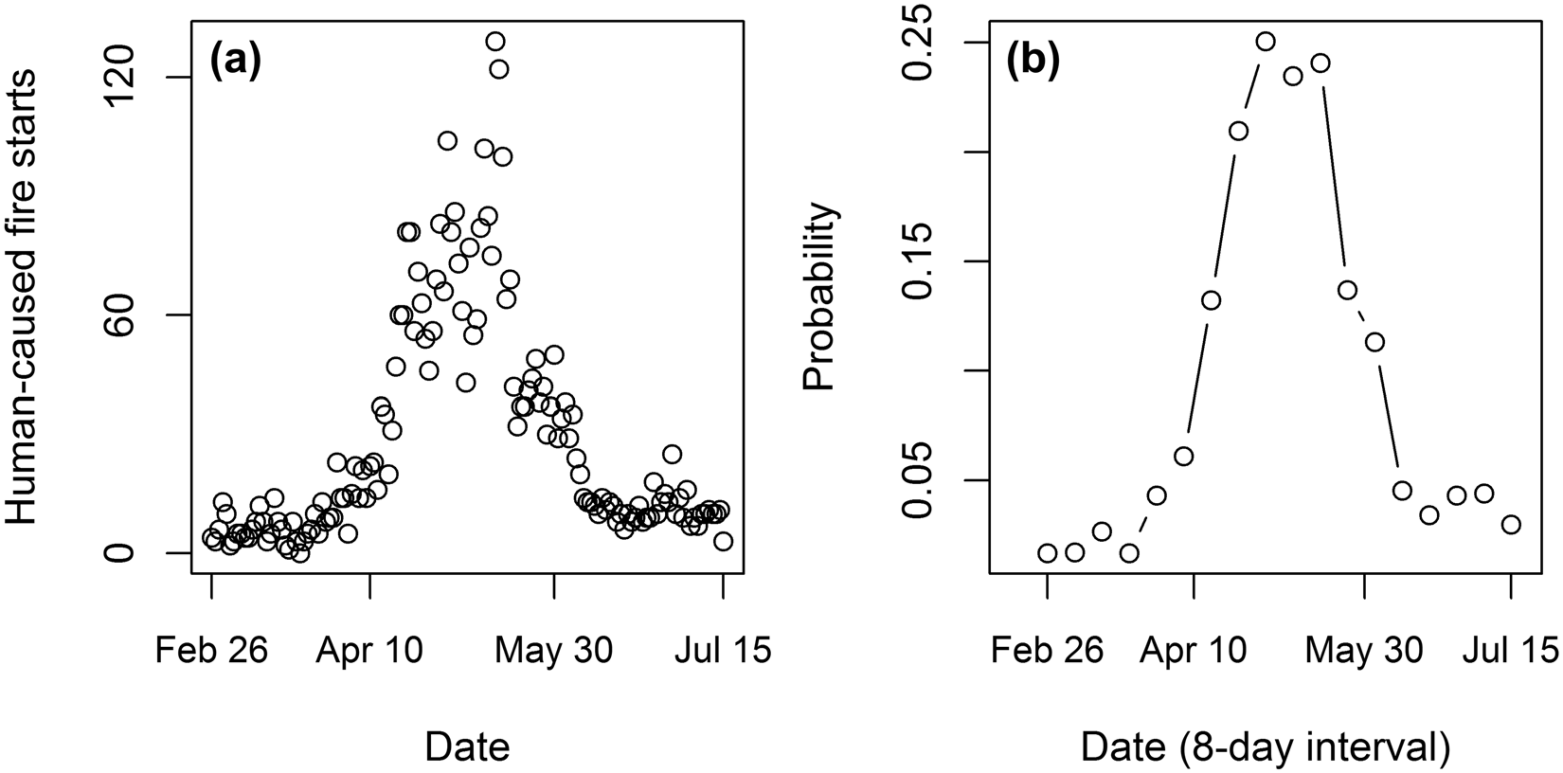
(**a**) Total number of human-caused wildfire starts by date between 2000 and 2014 and (**b**) the probability of a human-caused wildfire day modelled as a binomial process by eight-day intervals corresponding to the frequency of satellite observations of greenness.

Human-caused wildfire activity during the years 2000 and 2014 peaked in May (Fig. 2a). Days on which the probability of a human-caused wildfire day exceeded 20% ranged from May 1 to May 17 (Fig. 2b). Human-caused wildfires burned an average of 74,872 ha per year between 2000 and 2014, and approximately 95% of total area burned by human-caused wildfires across all years burned during the month of May.

**Figure 3:**
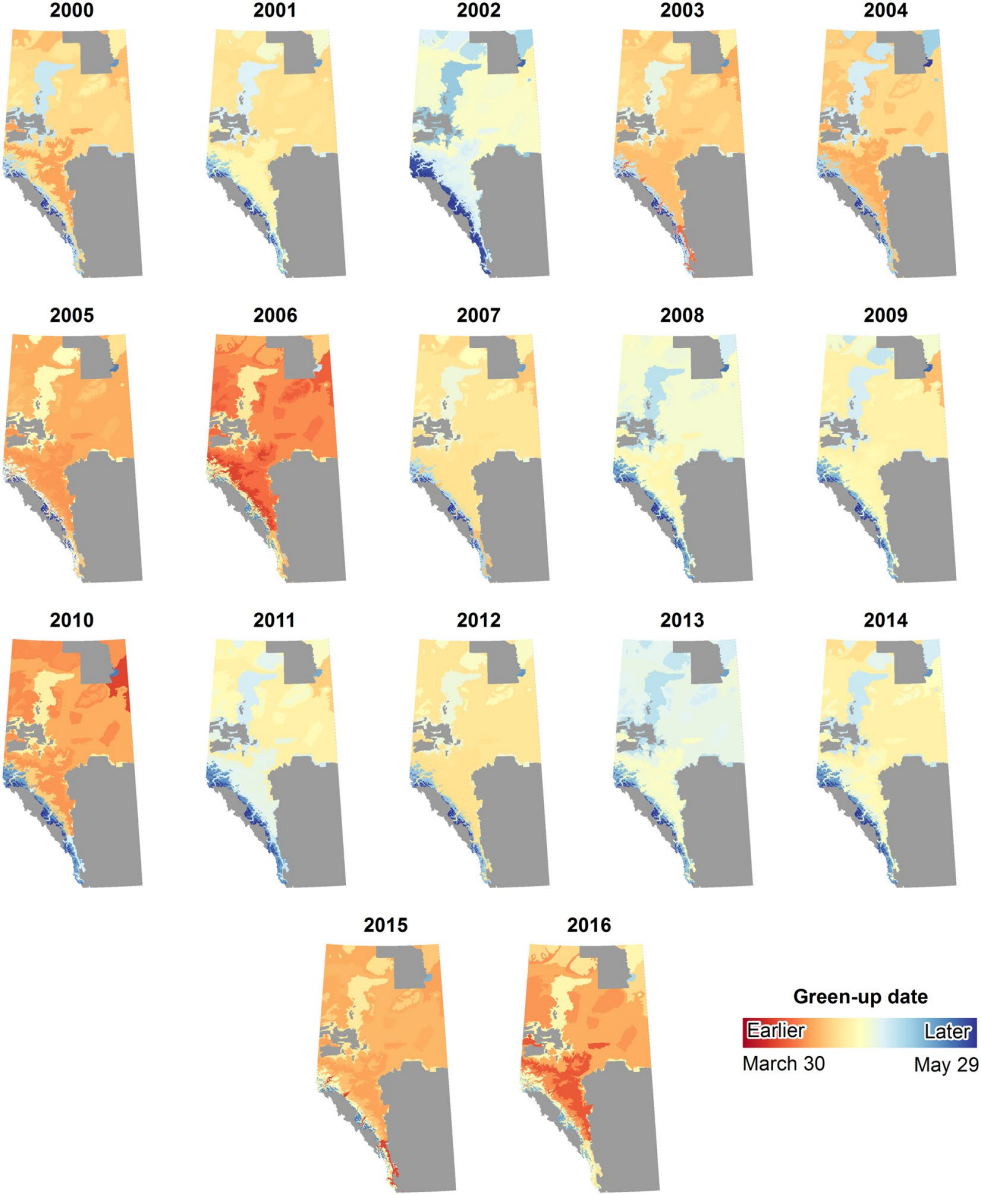
Green-up date for each natural sub-region modelled from half-maximum greenness for all years. Earlier dates represent earlier than average green-up and later dates represent later than average green-up relative to the entire wildfire protection area for all years (April 29). Maps created using ArcMap 10.5.1. (http://desktop.arcgis.com/en/arcmap/).

Vegetation green-up varied from year-to-year within each natural sub-region (Fig. 3). The Alpine and Subalpine natural sub-regions consistently showed the latest green-up dates across all years and natural sub-regions. The earliest observed green-up occurred on April 7, 2015 (day-of-year [DOY] 97) in the Montane natural sub-region, April 7, 2006 in the Upper Foothills natural sub-region, and April 7, 2010 in the Athabasca Plain natural sub-region. The latest observed green-up was on June 13, 2002 (DOY 164) in the Alpine natural sub-region. Much of the wildfire protection area was greener earlier in the season for the years 2005, 2006, 2010, 2015, and 2016 (Fig. 3). The years 2002, 2008, and 2013 showed that vegetation green-up was later in the season (Fig. 3).

**Figure 4:**
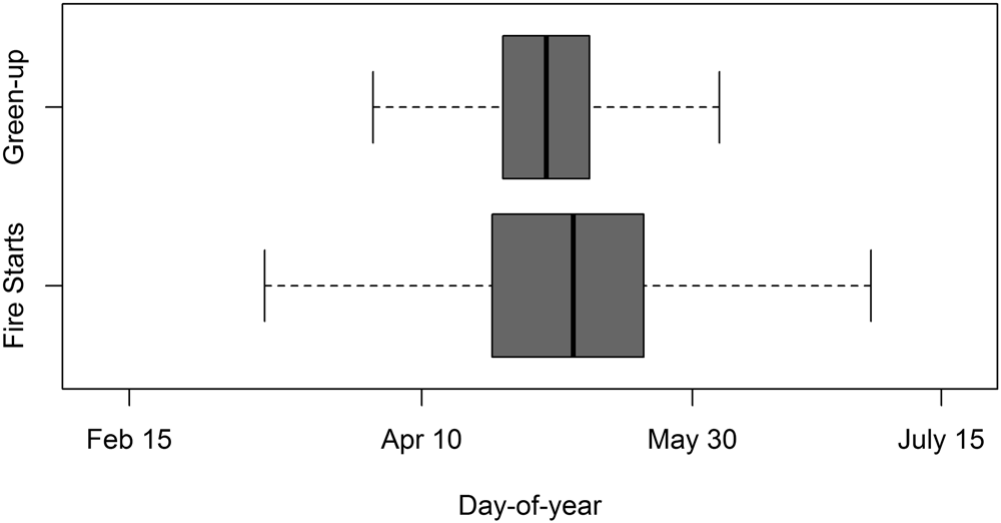
Barplot showing significant overlap in the interquartile ranges of the green-up predictions derived from normal greenness curves and human-caused wildfire starts during the spring burning window. The median green-up date was May 3 (day-of-year [DOY] 123) compared with May 8 (DOY 128) for human-caused wildfire ignitions.

Springtime human-caused wildfire activity was concomitant with satellite-observed green-up. Human-caused wildfire ignitions had a wider range in the season compared with green-up in the wildfire protection area. The interquartile ranges for vegetation green-up and human-caused wildfire ignitions overlapped completely for all years, confirming that wildfire activity peaked shortly following vegetation green-up during the spring burning window (Fig. 4). The median green-up date for all years was May 3 (DOY 123) compared with May 8 (DOY 128) for human-caused wildfire ignitions.

**Figure 5:**
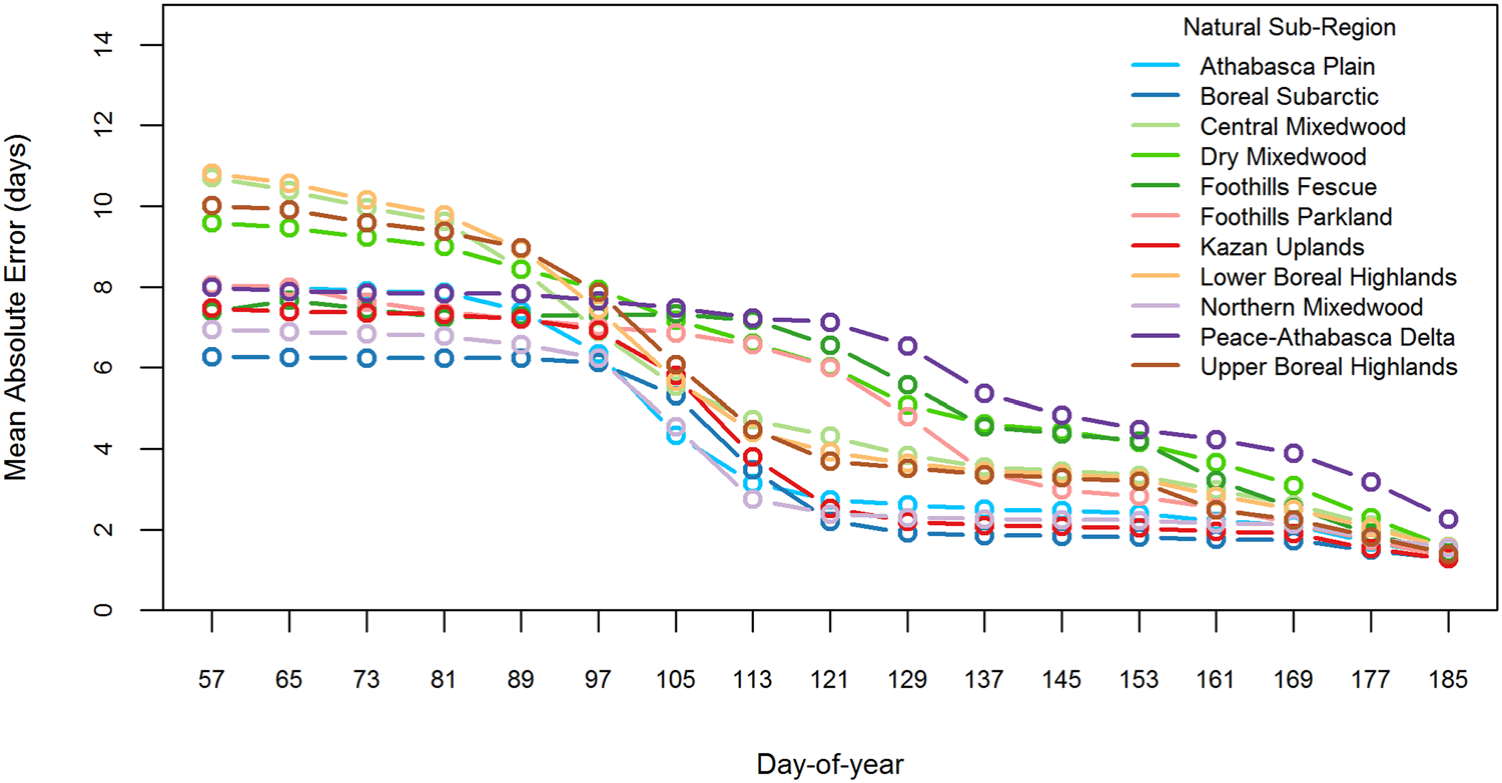
Forecasting the green-up day-of-year (DOY) was simulated for each year by successively adding greenness observations to the mean greenness curve and calculating the mean absolute error (MAE) within each natural sub-region. The natural sub-regions shown represent 76% of the wildfire protection area and the green-up DOY was forecasted to be within 10 days of the actual green-up DOY by March 22 (DOY 81).

**Figure 6:**
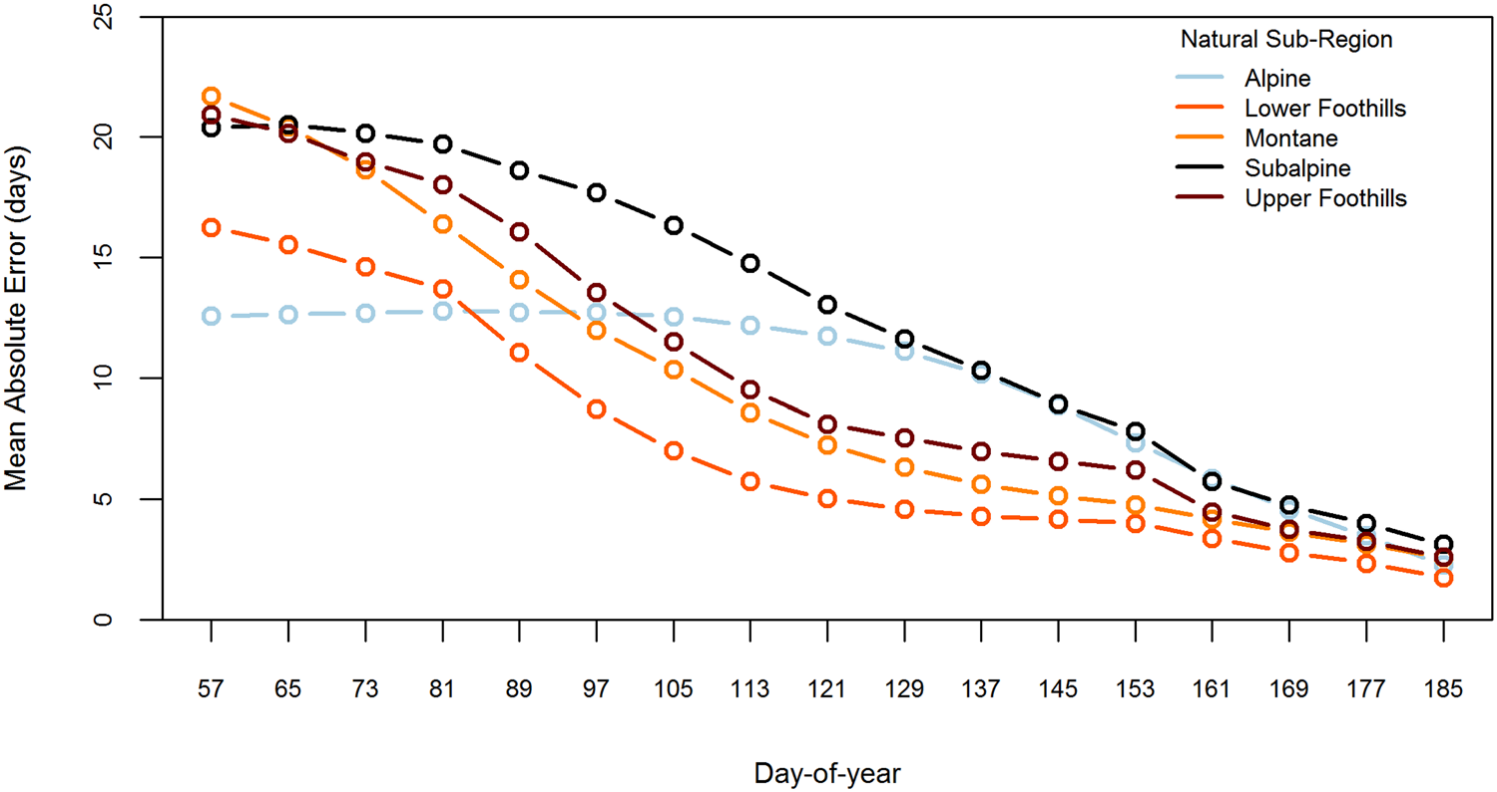
Forecasting the green-up day-of-year (DOY) was simulated for each year by successively adding greenness observations to the mean greenness curve and calculating the mean absolute error (MAE) within each natural sub-region. The natural sub-regions shown represent 24% of the wildfire protection area and the green-up DOY was forecasted to be more than 10 days from the actual green-up DOY by March 22 (DOY 81).

The mean absolute error (days) for the forecasted green-up date decreased in all natural sub-regions as more observations were made available in the season (Figs 5 and 6). By March 22 (DOY 81), vegetation green-up was forecasted to be within 10 days of the actual green-up date in any given year for 76% of the wildfire protection area (Fig. 5). A 10-day window of accuracy was not achieved until later in the season for the non-boreal natural sub-regions (Fig. 6): Alpine (May 25); Lower Foothills (April 7); Montane (April 23); Subalpine (May 25); and Upper Foothills (April 23).

**Figure 7:**
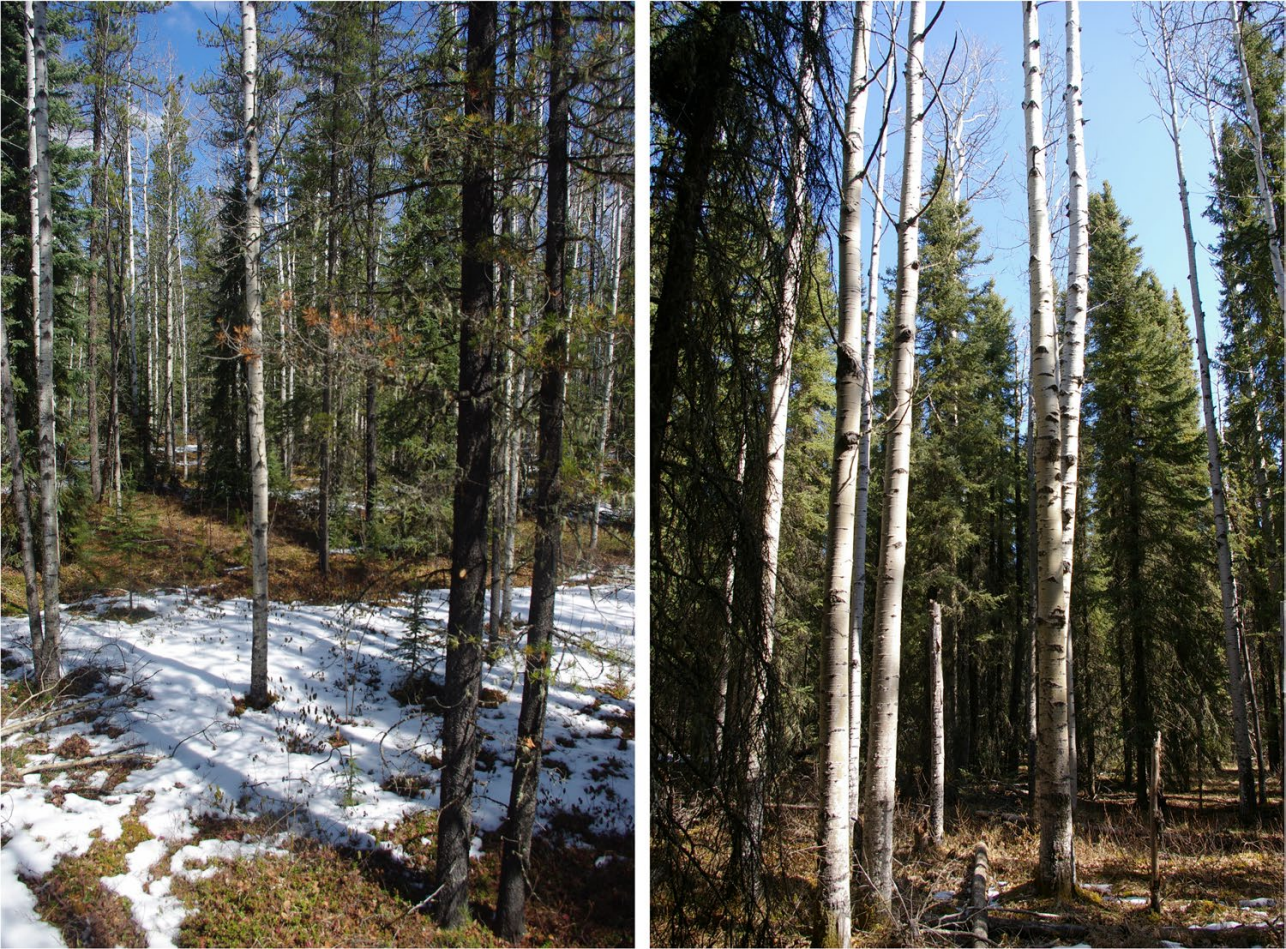
Leaf-off trembling aspen (*Populus tremuloides*) shown on clear dates (both photos taken on April 29, 2009) for two mixedwood sites around the mean predicted green-up date (April 27, 2009) for the Lower Foothills natural sub-region using the half-maximum greenness method. Photo by C.W. Bater.

**Figure 8:**
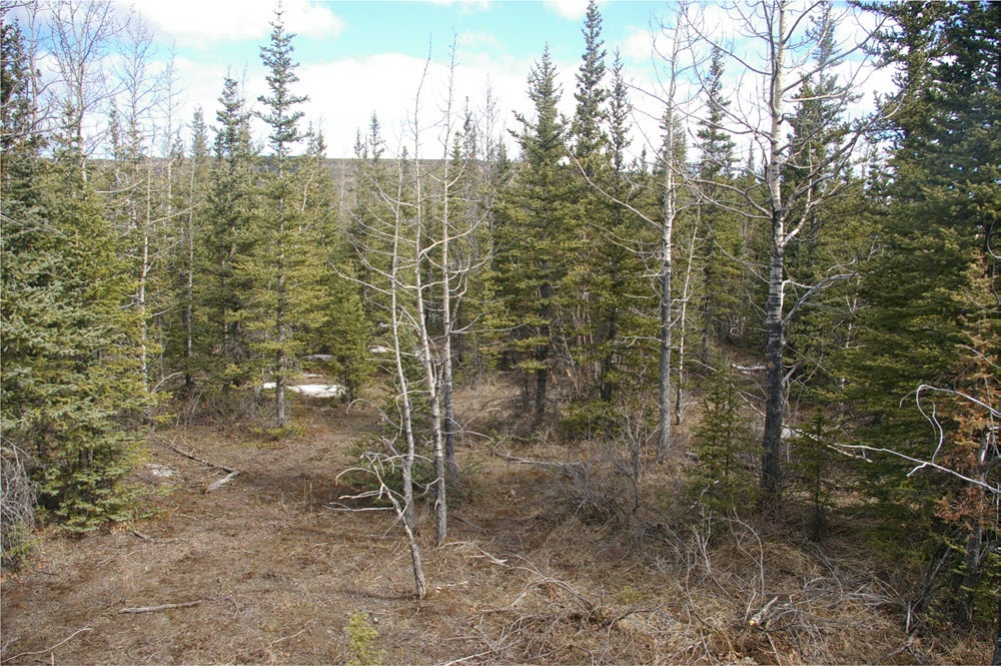
Mixedwood site in the Subalpine natural sub-region of Alberta. Leaf-off condition of balsam poplar (*Populus balsamifera*) shown on a clear day (May 7, 2009) with recent snow cover around the mean predicted green-up date (May 15, 2009) for the Subalpine natural sub-region using the half-maximum greenness method. Photo by C.W. Bater.

**Figure 9:**
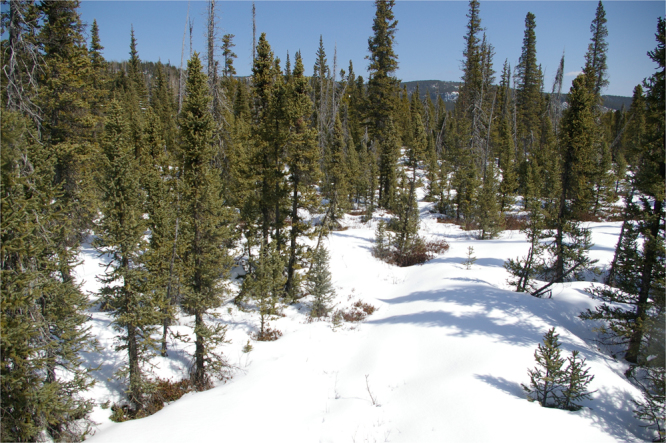
Coniferous site shown on a clear day (April 29, 2009) with recent snow cover around the mean predicted green-up date (April 28, 2009) for the Upper Foothills natural sub-region using the half-maximum greenness method. Photo by C.W. Bater.

From phenological camera observations within the Foothills and Subalpine natural sub-regions, it is apparent that leaf-off conditions in mixedwood stands were concomitant with the timing of peak human-caused wildfire activity in early May (Figs 7 and 8). In a coniferous site in the Alpine natural sub-region, changes in greenness were more subtle and consequently fine fuel moisture content was less obvious (Fig. 9). Snow cover was evident in all of the photos around the predicted green-up date, indicating possible interference in the signal-to-noise ratio for NDVI and may explain the high variability in predictions from year-to-year in these regions.

## Discussion

Globally, northern boreal forests are seeing an increase in industrial activity, including the establishment of road networks, oil and gas development, timber harvesting, and mining^39^. These activities have transformed historically remote boreal forests into anthropogenically-modified landscapes that pose increased risks for human-caused wildfire ignitions. As human uses of boreal forests increases in the near term, we can expect that the legacy of wildfire suppression from the beginning of the 20^th^ century may exacerbate persistent wildfire hazards around settlements and critical infrastructures.

The economic costs associated with wildfire suppression are recognized to be very high in the early months of the wildfire season and the variability of wildfire-fighting costs from year-to-year increases uncertainty for wildfire managers^40^. Additionally, increased human-caused wildfire activity can be expected to increase social costs such as displacement from evacuations and increased smoke concentrations near settlements and over large areas^41^. The wildfire that devastated the town of Fort McMurray, Alberta, in May 2016 was the costliest natural disaster in Canadian history and the second costliest wildfire globally, resulting in an estimated C$3.58 billion insurable damages^42^ and the mandatory evacuation of approximately 88,000 residents^43^. Additionally, the proximity of the Fort McMurray wildfire to one of the largest oil mining operations globally resulted in an estimated loss of 800,000 barrels per day due to production stoppages^44^. Thus, an economical early warning system is critical for forecasting wildfire spread potential and mitigating these socioeconomic costs in the wildland-urban interface from human-caused wildfires^40,45^. With the rapid increase in wildfire suppression activities in recent decades, the wildfire statistics in Alberta demonstrate that the spring burning window remains the most dangerous part of the year with respect to loss of property and infrastructure^40^.

The ability of wildfire ignitions to quickly spread in early spring is a well-known phenomenon attributed to a dip in foliar moisture and the drying of fine fuels^8^. These vegetation conditions contribute to increased wildfire frequency, area burned, and wildfire size during springtime. Historical and contemporary wildfire records show that the spring burning window results in increased wildfire activity between May and June, which coincide with observable rapid changes in the vegetation phenology using remote sensing observations from this study. The increasing popularity and use of inexpensive visible spectrum digital cameras offers notable potential for the monitoring and measurement of phenological events such as spring green-up^37,46^. Repeat photography allows for very fine temporal sampling, often at daily or hourly intervals, for monitoring vegetation. By mounting these camera systems on towers, data can be acquired at an intermediate scale of observation, providing a link between field-based observation methods and satellite-derived estimates. In addition, the cameras provide a visual link between satellite-observed greenness over larger spatial scales to field-based measurements and observations from local experts. Our results demonstrate that the phenological camera network utilized in this study provides evidence of leaf-off conditions in mixedwood sites during peak human-caused wildfire activity in early May. To be effectively integrated into a near-realtime monitoring and forecasting system, however, these remote cameras would require additional investment in wireless technology for downlinking the imagery.

Some challenges remain for accurately forecasting vegetation green-up in higher elevation areas with shorter growing seasons and less pronounced deciduous vegetation. In Alberta, these areas account for only 24% of the wildfire protection area (Fig. 6) and are sparsely populated (*i.e*., lower overall human-caused wildfire risk). The phenological camera observations demonstrated that green-up was reliably detected within mixedwood forests (Figs 7 and 8), underscoring the reliability of NDVI for observations with low signal-to-noise ratio. NDVI is quite sensitive to initial greening while leaf area index is below 2^47^, which is ideal for studying vegetation green-up. In sparser and coniferous forests occurring at higher elevations, the sensitivity of NDVI may outperform other vegetation indices that are less sensitive to lower leaf area index values. Additionally, MODIS is limited to the number of vegetation indices that may be calculated at 250 m spatial resolution because there are only two bands that are acquired at this scale. The signal-to-noise ratio may increase for NDVI with larger pixel sizes (e.g., 500 m, 1000 m) and this could possibly degrade the accuracy of a forecast especially for the higher elevation forests.

Simulation of the greenness curves from the end of winter to the end of vegetation green-up indicate that the vegetation green-up period was forecasted with 10-day accuracy for the majority of wildfire protected lands in Alberta by March 22 (DOY 81). This provides key evidence that vegetation greenness may be used as a reliable proxy for fine fuel moisture content. The eight-day composites limit the temporal precision available from the satellite data and introduce some uncertainty in the forecast. There is a fundamental trade-off between acquiring cloud-free satellite observations from MODIS and having sufficient temporal resolution to undertake the forecast on a near-weekly basis. Although in some cases a wildfire start date may be estimated, we report the probability of a wildfire day in terms of the 8-day MODIS intervals (Fig. 2). This procedure ensures that any uncertainty in the wildfire start date is minimized. For example, it would be very unlikely that a wildfire start date is off by more than 8 days because initial attack protocols are very successful and rely on timely reporting of wildfire starts. Despite this limitation, the early warning system was able to achieve 10-day accuracy very early in the wildfire season and the difference in timing of green-up and human-caused wildfire activity was only five days at the median. Moreover, satellite-observed greenness data are standardized and processed on a continual basis as observations become available. The early-warning system developed here offers significant benefits to wildfire managers for prioritizing the deployment of wildfire protection resources.
